# Quantitative proteomic analysis of enhanced cellular effects of electrochemotherapy with Cisplatin in triple-negative breast cancer cells

**DOI:** 10.1038/s41598-019-50048-9

**Published:** 2019-09-26

**Authors:** Lakshya Mittal, Uma K. Aryal, Ignacio G. Camarillo, Rodrigo M. Ferreira, Raji Sundararajan

**Affiliations:** 1 School of Engineering Technology, Purdue University, West Lafayette, IN 47907 USA; 2 Purdue Proteomics Facility, Bindley Bioscience Center, Purdue University, West Lafayette, IN 47907 USA; 3 Department of Biological Sciences, Purdue University, West Lafayette, IN 47907 USA; 4 Purdue Center for Cancer Research, Purdue University, West Lafayette, IN 47907 USA

**Keywords:** Proteomics, Breast cancer

## Abstract

Due to the lack of the three main receptors, triple negative breast cancer (TNBC) is refractive to standard chemotherapy. Hence, alternate therapies are needed. TNBCs utilize glycolysis, which heightens their growth, proliferation, invasiveness, chemotherapeutic resistance and poor therapeutic response. This calls for novel therapeutic strategies to target these metabolic vulnerabilities present in TNBC. Electroporation-mediated chemotherapy, known as electrochemotherapy (ECT) is gaining momentum as an attractive alternative. However, its molecular mechanisms need better understanding. Towards this, label-free quantitative proteomics is utilized to gain insight into the anticancer mechanisms of ECT using electrical pulses (EP) and Cisplatin (CsP) on MDA-MB-231, human TNBC cells. The results indicate that EP + CsP significantly downregulated 14 key glycolysis proteins (including ENO1, LDHA, LDHB, ACSS2, ALDOA, and PGK1), compared to CsP alone. EP + CsP caused a switch in the metabolism with upregulation of 34 oxidative phosphorylation pathway proteins and 18 tricarboxylic acid (TCA) cycle proteins compared to CsP alone, accompanied by the upregulation of proteins linked to several metabolic reactions, which produce TCA cycle intermediates. Moreover, EP + CsP promoted multiple pathways to cause 1.3-fold increase in the reactive oxygen species concentration and induced apoptosis. The proteomics results correlate well with cell viability, western blot, and qPCR data. While some effects were similar for EP, more comprehensive and long-lasting effects were observed for EP + CsP, which demonstrate the potential of EP + CsP against TNBC cells.

## Introduction

Breast cancer is the second major cause of cancer death (after lung cancer) in women. Annually, one million new patients are estimated to be diagnosed with breast cancer^1^. Triple negative breast cancer (TNBC) contributes to about 15–20% of these cases. TNBC is a heterogeneous phenotype of the breast cancer, which lacks the expression of estrogen (ER), progesterone (PR), and human epidermal growth factor receptor 2 (HER2) receptors^2^. It has an aggressive clinical course and poor prognosis^3^ with a short 5 year survival and increased 3 year recurrence rates^4^. There is a significantly increased likelihood of distant recurrence in TNBC (33.9%) compared to other breast cancers (20.4%)^3^. Aggressive and metastatic behavior of TNBC with distinct molecular signature and absence of targeted therapies pose a major challenge in the treatment. Recent reports suggest that TNBC harbor alterations in their metabolism, which correlate with the increased growth, proliferation, invasiveness, chemotherapeutic resistance and poor therapeutic response^4–6^. Particularly, TNBCs have elevated uptake and utilization of glucose and are highly dependent upon aerobic glycolysis^6,7^, while having disrupted mitochondrial function and oxidative phosphorylation (OXPHOS)^8,9^. The altered metabolism makes TNBC highly vulnerable to the inhibition of glycolysis, signifying that metabolic manipulation could be an effective strategy against TNBC. Consequently, the interest is growing in advancing new therapeutic strategies for TNBC patients when they cannot benefit from the current standard of treatment, including hormonal and trastuzumab therapies, because of the absence of target receptors^10^.

Cisplatin, the most potent platinum-based anticancer drug is commonly administered for many cancers including TNBC^11^. Minimizing the undesired side-effects, while maintaining Cisplatin’s potency and expanding its application to various cancer subtypes is of practical interest^12^. Cisplatin’s mechanisms of cellular uptake and efflux are not completely clear. Passive diffusion of Cisplatin takes place into the cells, where it interacts with various cellular components, including DNA to form platinum-DNA adducts by covalently binding to N7 positions of the purine bases^11^. Several pathways are activated by this platinum-DNA adduct formation, which mediate Cisplatin’s cytotoxicity. However, Cisplatin induces various adverse effects, including neurotoxicity, nephrotoxicity, and emetogenesis, limiting its application. Neurotoxicity, in particular, is a significant problem as excessive toxicity of Cisplatin can cause high-frequency hearing loss, tinnitus, and peripheral neuropathy^13^. Moreover, disruptions of off-target cellular components and pathways that are not involved in promoting tumor growth and metastasis are of great concerns. Improving drug’s effectiveness at low concentration is important for reducing drug related toxic side effects, and for developing chemotherapy as a front-line cancer therapy. Cisplatin is also a poorly permeant molecule and one of the ways to address this problem is to improve drug permeabilization to the cells.

Electrochemotherapy (ECT) involves the local application of high intensity, short duration voltage pulses to tumor tissues and cancer cell lines to enhance the uptake of chemo drugs, by rendering the normally impermeable or poorly permeable plasma cell membranes permeable^14^. This results in increased intracellular concentrations of the drug, causing significant cell death. There is no inflammatory reaction as the treatment is confined to the area where the electric field is applied. ECT is shown to increase the Cisplatin cytotoxicity up to 80–100-fold with reduced side-effects^15,16^, and it is now increasingly used to achieve several fold enhancement in the cellular uptake^17–20^. Increased cytotoxicity of Cisplatin and Bleomycin by ECT has also been demonstrated in several studies in human cell lines, rodent and other animal models^18,20^.

ECT is applicable to all histologies of cancers and it has been used clinically to treat various melanomas, carcinomas, sarcomas, and many other types of cancer^21–30^. ECT is also used for treating brain^26^, bone^27^ and liver^28^ metastases. In 2014, the National Health Services in England, Wales, Scotland and Northern Ireland were issued a full guidance on ECT for primary squamous cell and basal cell carcinomas by the National Institute for Health and Excellence (NICE)^25^. In a multi-institutional study, Campana *et al*. treated 376 patients with breast carcinoma, squamous cell and basal cell carcinoma, Kaposi sarcoma, soft tissue sarcoma, melanoma, and others using ECT^21^. In a multicenter cohort study, breast cancer skin metastases in 125 patients was treated with ECT with overall response rate of 90.2% after 2 months and 58.4% complete response (CR) rate, leading to durable local control in patients who had CR^29^. In another case, Campana *et al*. treated 84 patients with multiple, recurring or locally advanced basal cell carcinoma (BCC), ineligible of standard therapy treatment; when treated with Bleomycin-ECT resulted in tumor eradication in 50% and 63% of patients, after single or two cycles of ECT^22^. A long-lasting CR of 77% was obtained in patients treated with Cisplatin-ECT at Cisplatin doses which show no anti-cancer activity and side effects on their own, compared to 19% obtained with Cisplatin alone with similar doses^20,31^. ECT is now included in several national and international guidelines for primary skin cancer and cutaneous metastases^25,30^.

The effects of ECT are driven by multiple signaling pathways that involve a collection of genes, which encode proteins with different functions ranging from cell surface receptors to transcription factors^32^. Modern genomics and proteomics techniques provide us opportunity to discover new proteins and pathways involved in the cellular signaling network in response to ECT.

High throughput, Mass Spectrometry (MS)-based proteomics enables system-level analysis of thousands of proteins in complex biological samples^33,34^. Bioinformatic analysis of the expressed proteome and their known and predicted molecular interactions can be used to determine functional roles of each expressed protein. Here, we performed a comprehensive and label-free quantitative proteomics study of the human adenocarcinoma epithelial TNBC cells (MDA-MB-231) treated with Cisplatin only (CsP), and electrical pulses only (EP), and EP with Cisplatin (EP + CsP). We identified hundreds of differentially expressed proteins that defines molecular mechanisms behind the cellular responses of MDA-MB-231 cells to these treatments. The dataset represents a valuable resource for mechanistic studies. The systemic data could build a foundation for an improved mechanistic understanding of Cisplatin’s cytotoxicity as a potent anticancer drug for TNBC.

## Results

### Cell viability measurement

**Figure 1:**
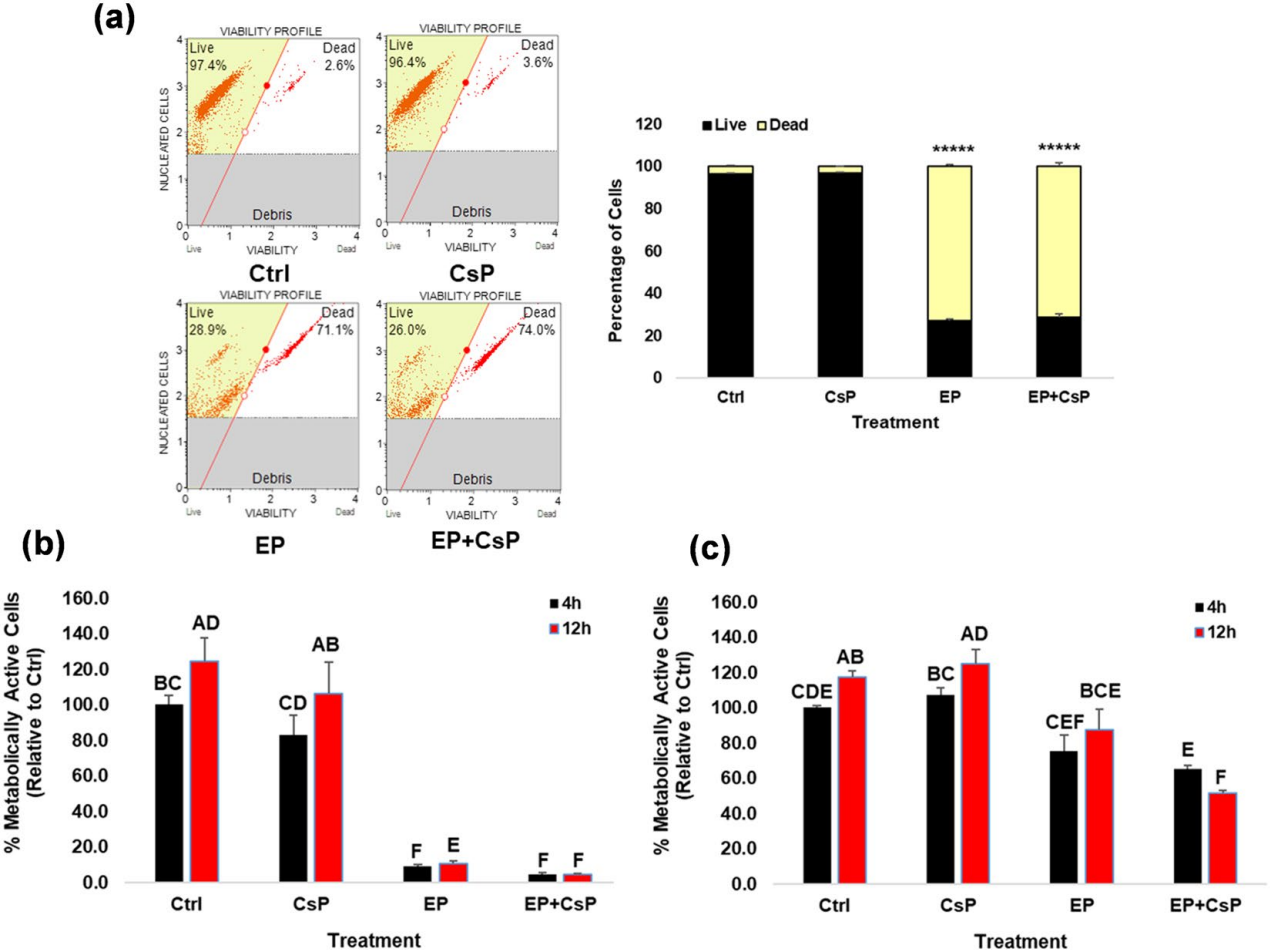
(**a**) The MDA-MB-231 cell viability at 4 h of treatments: Control (Ctrl), Cisplatin (CsP), electrical pulses (EP), and EP + Cisplatin (EP + CsP). Cells were incubated for 5 mins at room temperature (RT) with Muse™ Count & Viability reagent, which differentially stains viable and non-viable cells based on their permeability to the two DNA binding dyes present in the reagent. Cells were run on Muse^®^ Cell Analyzer. *****P < 0.000005-significantly different from Ctrl. (**b**,**c**) The percentage of metabolically active MDA-MB-231 cells (**b**) and MCF10A cells (**c**) at 4 h and 12 h of treatments. Cells were incubated for 4 h and 12 h with RealTime-Glo^TM^ Cell Viability reagent, which measures the ATP-independent reducing potential of cells to quantify metabolically active cells relative to the 4 h Ctrl. The same letter or the same groups of letters indicate that they are not significantly different. The different letters or different group of letters indicate that they are significantly different (P < 0.05).

Figure 1a shows the viability of MDA-MB-231 cells at 4 h after treatments. The viable and non-viable cells were quantified using flow cytometry based on cell membrane permeability using two DNA binding dyes. The control cells (Ctrl) and the CsP treated cells had high viabilities of 96.3% and 96.9%, indicating poor cell death, while the EP and EP + CsP cells had lower viabilities of 27% and 28.9%, indicating no significant differences in cell viabilities of these two treatments.

Figure 1b shows the metabolic activity of MDA-MB-231 cells at 4 h and 12 h of the treatments by quantifying ATP-independent substrate reducing potential. The results were normalized with the metabolic activity of Ctrl cells at 4 h (100%). The metabolic activity of Ctrl cells increased to 125% at 12 h. In the CsP treated samples, 83% and 106% of cells were metabolically active at 4 h and 12 h, respectively, showing no significant reduction in the metabolic activity (Fig. 1b). The EP treatment decreased the metabolic activity to 8.8% initially at 4 h, but the live cells revived back as indicated by a continued increase in the metabolic activity to 10.6% at 12 h and to 16% at 60 h (Table S1). For EP + CsP treatment, the fraction of metabolically active cells decreased significantly to 4.5% and 4.2% at 4 h and 12 h, respectively. The metabolic activity for EP + CsP treated cells showed a time dependent decrease as it reduced to 4% at 60 h (Table S1), which highlights a sustained effect of the EP + CsP treatment, contrary to EP.

Since *in vivo* application will involve both healthy and cancerous cells, we assessed the metabolic activity of non-tumorigenic mammary epithelial MCF10A cells (Fig. 1c). For EP treatment, the metabolic activity of MCF10A cells were 75% and 88% at 4 h and 12 h, respectively. For EP + CsP treatment, 65% and 51% of MCF10A cells were metabolically active at 4 h and 12 h, respectively. These results indicate that the effects of EP + CsP are specific to TNBC cells, as MCF10A cells maintained a significantly higher metabolic activity, compared to MDA-MB-231 cells.

### Overview of proteome analysis

**Figure 2:**
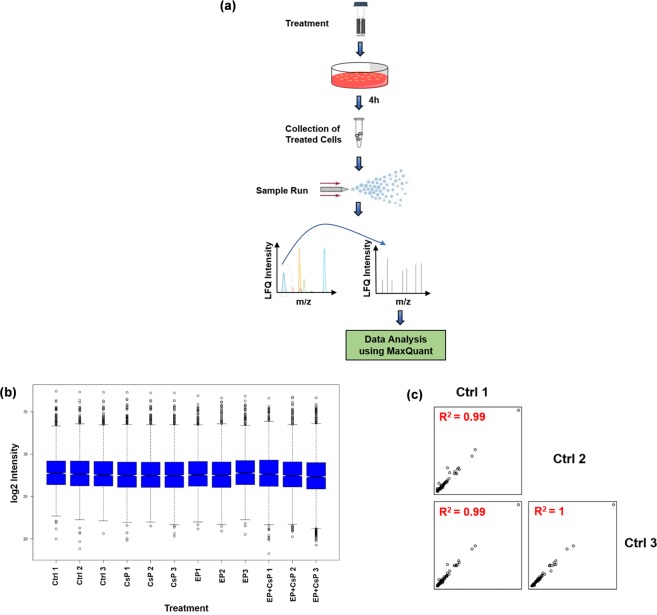
Proteomic workflow. (**a**) Control and treated cells were lysed in urea and analyzed by LC-MS/MS for protein identification and quantification. (**b**) Boxplot of biological replicates of each treatment group. (**c**) Correlation plots of three biological replicates of the control sample. R^2^ values are consistent and are noticeably higher between the replicates.

Figure 2a shows the experimental workflow for proteome analysis. LC-MS/MS data were collected using Q Exactive Orbitrap HF hybrid MS coupled with the UltiMate^TM^ 3000 RSLCnano HPLC system. The tandem mass spectra were searched against the UniProt human protein database. Proteins identified in at least two of the three biological replicates at 1% FDR and with at least 2 MS/MS (spectral) counts were considered for further analysis, resulting in unambiguous identification of 2867 proteins/protein families from 39622 peptides (Tables S2 and S3). Relative abundances of proteins in each sample were determined by label-free quantitation using LFQ values. Consistency of LFQ intensity is critical for the accurate measurement of protein abundances across multiple samples. The Boxplot (Fig. 2b) shows that the median and interquartile range of the triplicate samples were similar for each treatment, indicating the consistency of LC-MS/MS measurements among the replicates. To further establish an experimentally supported inventory of differentially expressed proteins, we also measured correlations of protein intensities in triplicates of each treatment. As shown in Fig. 2c, we obtained a coefficient of determination (R^2^) value of 0.99 for the pairwise comparisons of the Ctrl samples. Similar R^2^ values were observed for other treatments as well (data not shown). The high R^2^ values indicate high correlation and quality of the proteomics experiments.

**Figure 3:**
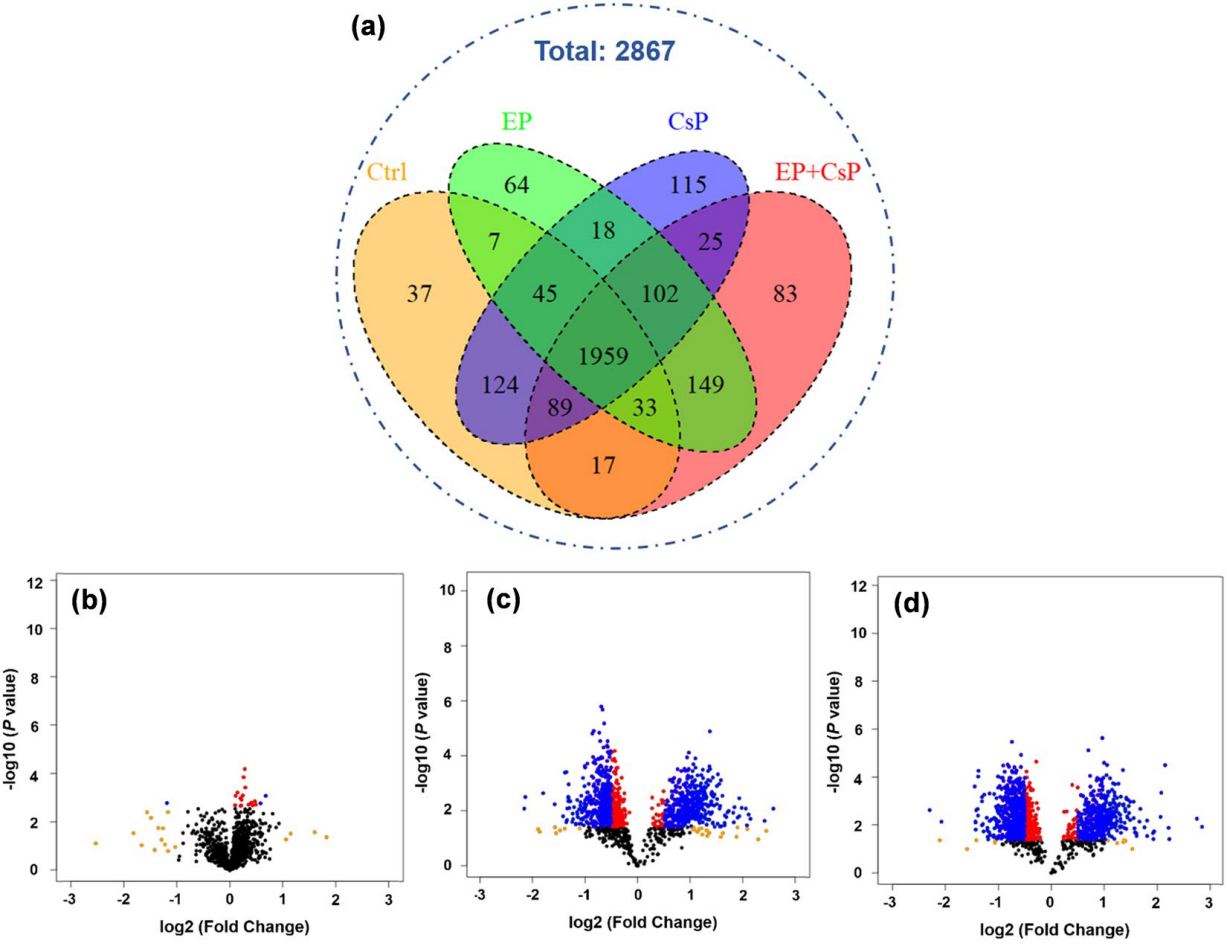
(**a**) Venn diagram showing the distribution of proteins in the three treatment groups. (**b–d**) Volcano plots showing the variation in log10 (P value) with log2 (fold change) for various pairwise comparisons: (**b**) CsP vs Ctrl, (**c**) EP + CsP vs Ctrl, and (**d**) EP + CsP vs CsP. Color map; black: no-significance (P > 0.05), red: significantly changed but not regulated (P < 0.05 and |log2 (fold change)| < 0.5), blue: significantly regulated (P < 0.05 and |log2 (fold change)| > 0.5).

We used LFQ values and MS/MS counts (LFQ ≠ 0 and MS/MS ≥ 2 for at least two of the three replicates) to classify the number of common and unique proteins present in each treatment. We identified 2311 proteins in control, 2477 in CsP, 2377 in EP, and 2457 in EP + CsP treated cells, of which 1959 (84.8% of the control) were commonly expressed in all three samples (Fig. 3a, Table S3). The number of uniquely identified proteins varied from 37 in Ctrl to 115 in CsP, 64 in EP, and 83 in EP + CsP samples. More proteins were identified in CsP than in the EP + CsP (2477 versus 2457) with more unique proteins, suggesting significant impact of ECT on cellular pathways through the increase or through the loss of many protein expressions (i.e., 302 proteins found in CsP were not found in EP + CsP).

### Analysis of differentially expressed proteins

**Figure 4:**
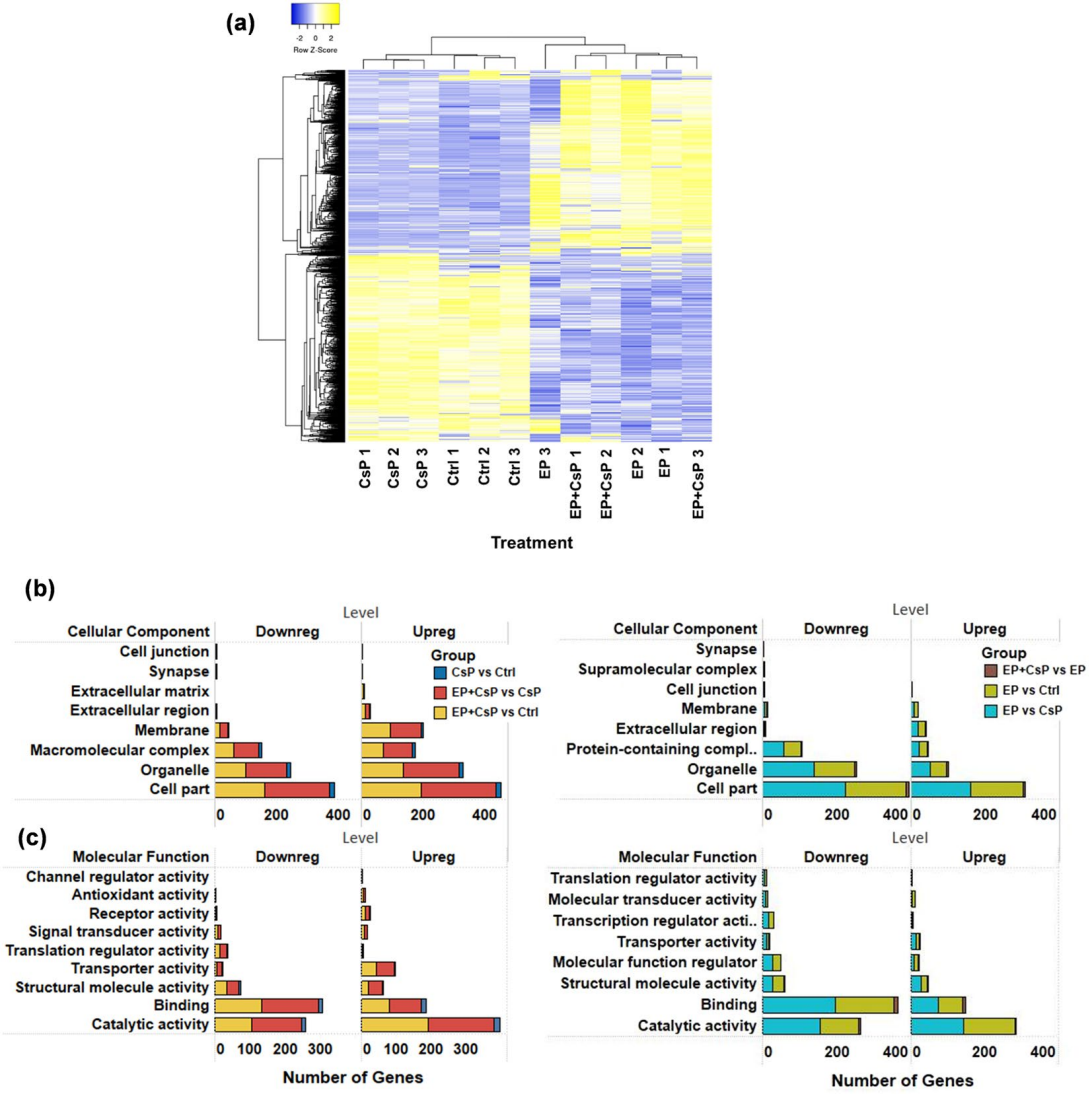
(**a**) Heatmap showing the expression levels of significantly regulated proteins in different treatments. LFQ intensities of proteins were clustered using Average Linkage method and Kendall’s Tau distance measurement method. Upregulated and downregulated proteins are represented with yellow and blue colors, respectively. (**b,c**) Localization (**b**) and functional classification (**c**) of significantly regulated proteins in various pairwise comparisons: CsP vs Ctrl, EP + CsP vs CsP, EP + CsP vs Ctrl, EP + CsP vs EP, EP vs Ctrl, and EP vs CsP.

The LFQ values of the common proteins in all four groups (1959) and common in two groups and unique in each group were used to perform statistical analysis and to detect significant differentially expressed proteins. Compared to the Ctrl, 46 proteins in the CsP, 364 proteins in the EP, and 512 proteins in the EP + CsP were upregulated (P < 0.05) (Table S4). Similarly, 36 proteins in the CsP, and 388 proteins in the EP and EP + CsP were downregulated (P < 0.05) (Table S4). When compared to CsP, 547 proteins were upregulated and 507 were downregulated in the EP + CsP treatment. Compared to EP, 13 proteins were upregulated and 16 were downregulated in the EP + CsP treatment. All proteins quantified in each treatment were visualized in Volcano plots depicting protein abundance changes between CsP versus Ctrl (Fig. 3b), EP + CsP versus Ctrl (Fig. 3c), and EP + CsP versus CsP (Fig. 3d). The differentially expressed proteins were clustered and visualized as a heatmap (Fig. 4a). Proteins within each treatment are clustered together and show consistent expression patterns. Between treatments, clusters of proteins for the Ctrl and CsP treatments are closer than the cluster of proteins for the EP and EP + CsP treatments, indicating the difference in protein expression induced by EP and EP + CsP treatments.

Further, we classified differentially expressed proteins using gene ontology (GO) annotation analysis for cellular component and molecular functions (Fig. 4b,c). EP affected a large number of proteins in the extracellular region, as well as in other cellular components, such as protein containing complexes, cell part, organelle (Fig. 4b). For EP + CsP, more proteins in membrane, cell part, organelle and macromolecular complexes assembly were upregulated. Molecular function analysis showed higher representation of proteins related to catalytic activity, binding, and structural molecule activity for both EP + CsP and EP (Fig. 4c). However, proteins in molecular function regulator, transcription regulator activity, and molecular transduction activity were uniquely affected for EP, and more proteins related to signal transduction, antioxidant activity, and receptor activity were uniquely affected for EP + CsP. A number of proteins involved in the translational activity were downregulated and more proteins involved in the transport activity were upregulated in the EP + CsP treatment compared to CsP and Ctrl treatments.

**Figure 5:**
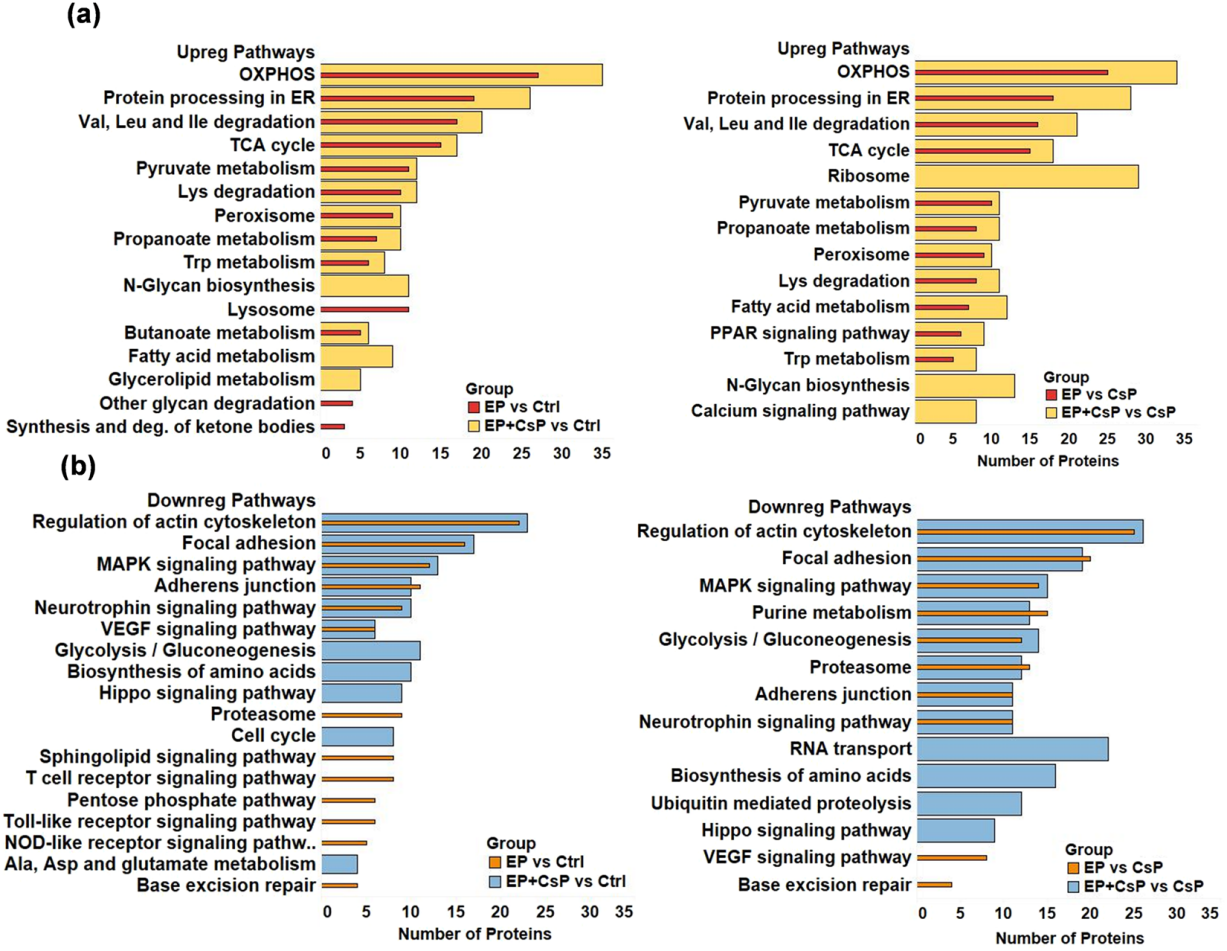
Key enriched pathways for significantly upregulated (**a**) and downregulated (**b**) proteins in different treatment comparisons using DAVID 6.8. The full list of enriched pathways is presented in Supplementary Fig. S1. Abbreviations - Ala: Alanine; Asp: Aspartate; Deg: Degradation; Isoleucine: Ile; Leu: Leucin, Lys: Lysine; Trp: Tryptophan; Val: Valine.

Analysis of KEGG pathways for upregulated proteins (Fig. 5a) showed enrichment of OXPHOS, tricarboxylic acid (TCA) cycle, fatty acid metabolism, aromatic amino acid (tryptophan) metabolism, calcium and PPAR signaling in EP + CsP and EP as compared to the Ctrl or CsP. However, the number of upregulated proteins in these pathways were lower for EP compared to EP + CsP, indicating the additional effect of CsP with EP.

The lysosome, other glycan degradation, synthesis and degradation of ketone bodies, and ECM-receptor interaction pathways were upregulated only in EP, but N-Glycan biosynthesis, fatty acid metabolism, fatty acid elongation, glycerolipid metabolism, biosynthesis of unsaturated fatty acids, calcium signaling, and ribosome pathways were upregulated only in EP + CsP compared to the Ctrl or CsP (see Fig. S1a for more information). The expression of glutaminase (GLS) increased in EP + CsP and EP treatments compared to the Ctrl and CsP.

In contrast, pathways involved in cell proliferation, differentiation and migration were downregulated for both EP + CsP and EP (Fig. 5b). This included several signaling pathways, such as MAPK signaling, neurotrophin signaling, and VEGF signaling. The pathways, such as hippo signaling, and biosynthesis of amino acids were only enriched for downregulated proteins in EP + CsP from Ctrl or CsP. On the other hand, the pathways, such as, T cell receptor signaling, Toll-like receptor signaling, NOD-like receptor signaling, base excision repair, estrogen signaling, and pentose phosphate pathway were only enriched for proteins downregulated in EP from Ctrl or CsP.

The cell cycle pathway was downregulated only in EP + CsP, but base excision repair, the pathway involved in the DNA repair throughout the cell cycle was downregulated only in EP. This indicates that presence of CsP is required to impact the cell cycle, and EP alone only slows down the DNA repair process, which is also consistent with the anti-proliferative effect of CsP, which induces cell cycle arrest in cancer cells^35^. An important protein, proliferating cell nuclear antigen (PCNA), which is involved in the repair of DNA damage caused by CsP was significantly downregulated in EP + CsP and EP treatments compared to the CsP (EP + CsP vs CsP - ↓2×, P = 0.0003; EP vs CsP - ↓1.7×, P = 0.0071) and Ctrl (EP + CsP vs Ctrl -↓1.8×; P = 0.0001; EP vs Ctrl - ↓1.5×, P = 0.012). The effect of EP + CsP were larger on PCNA compared to EP, and may help overcome the CsP resistance, as EP with CsP may increasingly stabilize CsP induced genomic DNA damage^36^.

Additionally, downregulated proteins included those in the regulation of actin cytoskeleton, focal adhesion, proteoglycans in cancer, purine metabolism, glycolysis/gluconeogenesis, amino acid biosynthesis, proteasomes, and ubiquitin mediated proteolysis. We observed downregulation of glycolysis pathway with 11 and 14 proteins in EP + CsP treatment compared to the Ctrl and CsP, respectively. For the EP treatment, while the glycolysis was not enriched for downregulated proteins from the Ctrl, but compared to the CsP, 12 downregulated proteins were enriched in glycolysis. For EP + CsP and EP treatments, the glucose metabolism proteins, notably fructose-bisphosphate aldolase A (ALDOA), alpha-enolase (ENO1), phosphoglycerate kinase 1 (PGK1), and lactate dehydrogenase isoforms A (LDHA) and B (LDHB) were all downregulated, compared to Ctrl and CsP, and acyl-CoA synthetase short-chain family member 2 (ACSS2), compared to CsP (Table S6).

**Figure 6:**
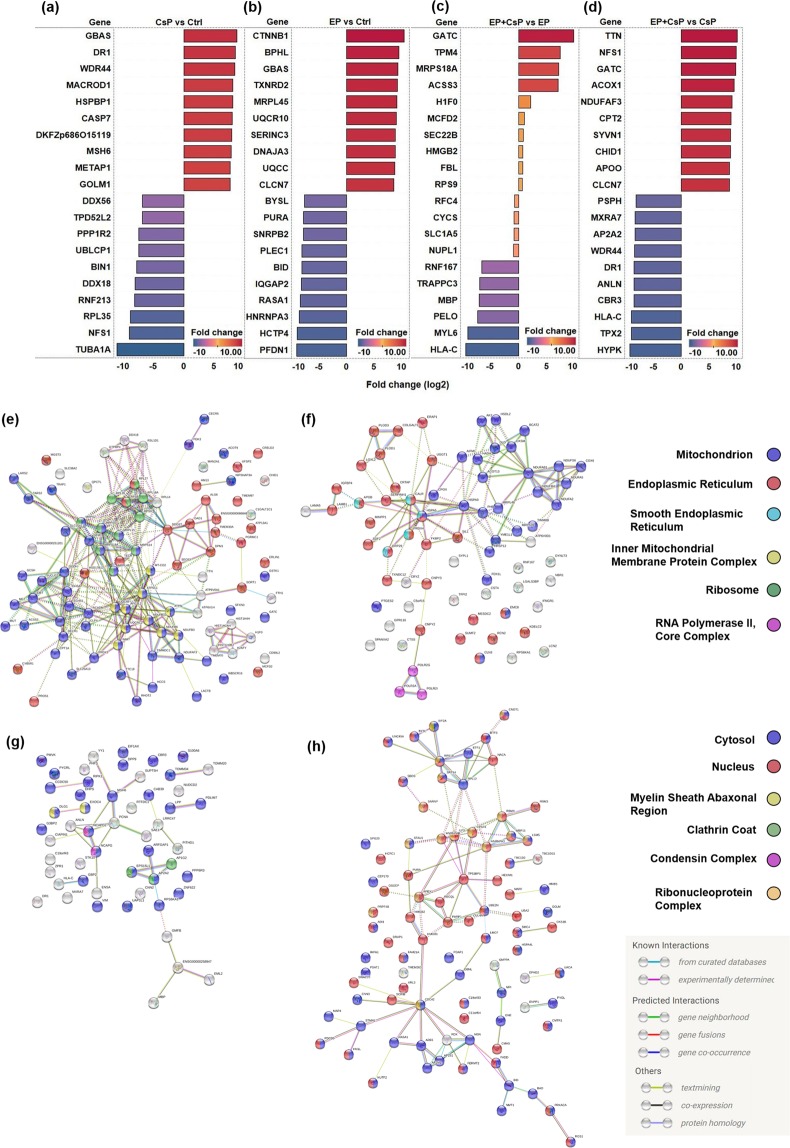
(**a**–**d**) The expression levels (log2 fold change) of top-10 upregulated and downregulated proteins in different comparison groups: (**a**) CsP vs Ctrl, (**b**) EP vs Ctrl, (**c**) EP + CsP vs EP, (**d**) EP + CsP vs CsP. (**e–h**) The String Interaction analysis of the most significantly regulated proteins (|fold change| ≥ 2) but uniquely regulated in EP + CsP or EP, compared to CsP (**e**) Upregulated in EP + CsP vs CsP, (**f**) Upregulated in EP vs CsP, (**g**) Downregulated in EP + CsP vs CsP, (**h**) Downregulated in EP vs CsP. EP + CsP and EP protein expressions were compared to CsP and highly significantly regulated (|fold change| ≥ 2) proteins were identified: 339 in EP + CsP and 348 in EP. Out of these proteins, those commonly regulated in EP + CsP and EP were filtered out to obtain uniquely regulated proteins: 154 in EP + CsP and 164 in EP. These proteins were uploaded to STRING^76^ to visualize the interaction and functional enrichment among these unique proteins with minimum required interaction score as medium confidence (0.4) and kmeans clustering (3 clusters). The nodes represent proteins, with color representing their localization. The colored nodes represent query proteins and first shell of interactions and white nodes represent second shell of interaction. Edges represent protein-protein associations. EP + CsP upregulated more proteins in the mitochondrion, but EP upregulated more proteins in the endoplasmic reticulum. In the downregulated proteins, EP + CsP affected more proteins in the cytosol, while EP affected a large number of proteins in the nucleus.

Figure 6(a–d) shows the list of top 10 upregulated and downregulated proteins and their expression levels (log2 fold change) in 4 different pairwise comparisons (CsP vs Ctrl; EP vs Ctrl, EP + CsP vs EP, EP + CsP vs CsP). The NipSnap homolog 2 (GBAS) was the most upregulated protein with a log2 fold change of 8.71 in CsP and catenin beta-1 (CTNNB1) was the most upregulated protein in EP with a log2 fold change of 10.42, compared to the Ctrl (Fig. 6a,b). Similarly, in the EP + CsP treatment glutamyl-tRNA (Gln) amidotransferase subunit C, mitochondrial (GATC) with a log2 fold change of 9.71, and titin (TTN) with a log2 fold change of 9.99 were the most upregulated proteins, compared to the EP and CsP treatment, respectively. (Fig. 6c,d).

The tubulin alpha-1A chain (TUBA1A) was the most downregulated protein with a log2 fold change of 10.99 in CsP and prefoldin subunit 1 (PFDN1) was the most downregulated protein with a log2 fold change of 9.07 in EP, compared to the Ctrl (Fig. 6a,b). Similarly, for the EP + CsP treatment, MHC class I antigen (HLA-C) with a log2 fold change of 9.27, and huntingtin-interacting protein (HYPK) with a log2 fold change of 9.20 were the most significantly downregulated proteins compared to the EP and CsP treatment, respectively (Fig. 6c,d).

Among the 29 significantly regulated proteins for EP + CsP from EP (Table S5), the key proteins were replication factor C subunit 4 (RFC4), Ras GTPase-activating protein-binding protein 2 (G3BP2), radixin (RDX), 40 S ribosomal protein S9 (RPS9), 28S ribosomal protein S18a, mitochondrial (MRPS18A). Intrestingly, high MS/MS spectral counts were observed for DnaJ homolog subfamily C member 3 (DNAJC3), amino acid transporter (SLC1A5), cytochrome c (CYCS), rRNA 2-O-methyltransferase fibrillarin (FBL), Ferritin (FTH1), Myosin regulatory light polypeptide 9 (MYL9), Mitochondrial import receptor subunit TOM34 (TOMM34), indicating high abundance of these differentially expressed proteins in EP + CsP compared to EP. A String interaction enrichment analysis (Fig. 6(e–h)) shows uniquely regulated proteins for EP + CsP and EP compared to CsP. There were more upregulated proteins in the mitochondrion for EP + CsP, while EP had more proteins in the endoplasmic reticulum (Fig. 6e,f). In the case of downregulated unique proteins, EP + CsP affected more cytosolic proteins, while a large number of nucleus proteins were downregulated by EP (Fig. 6g,h). These results specifically illustrate the differences in mechanism of EP + CsP from EP in MDA-MB-231 cells.

### Validation of proteomics results

**Figure 7:**
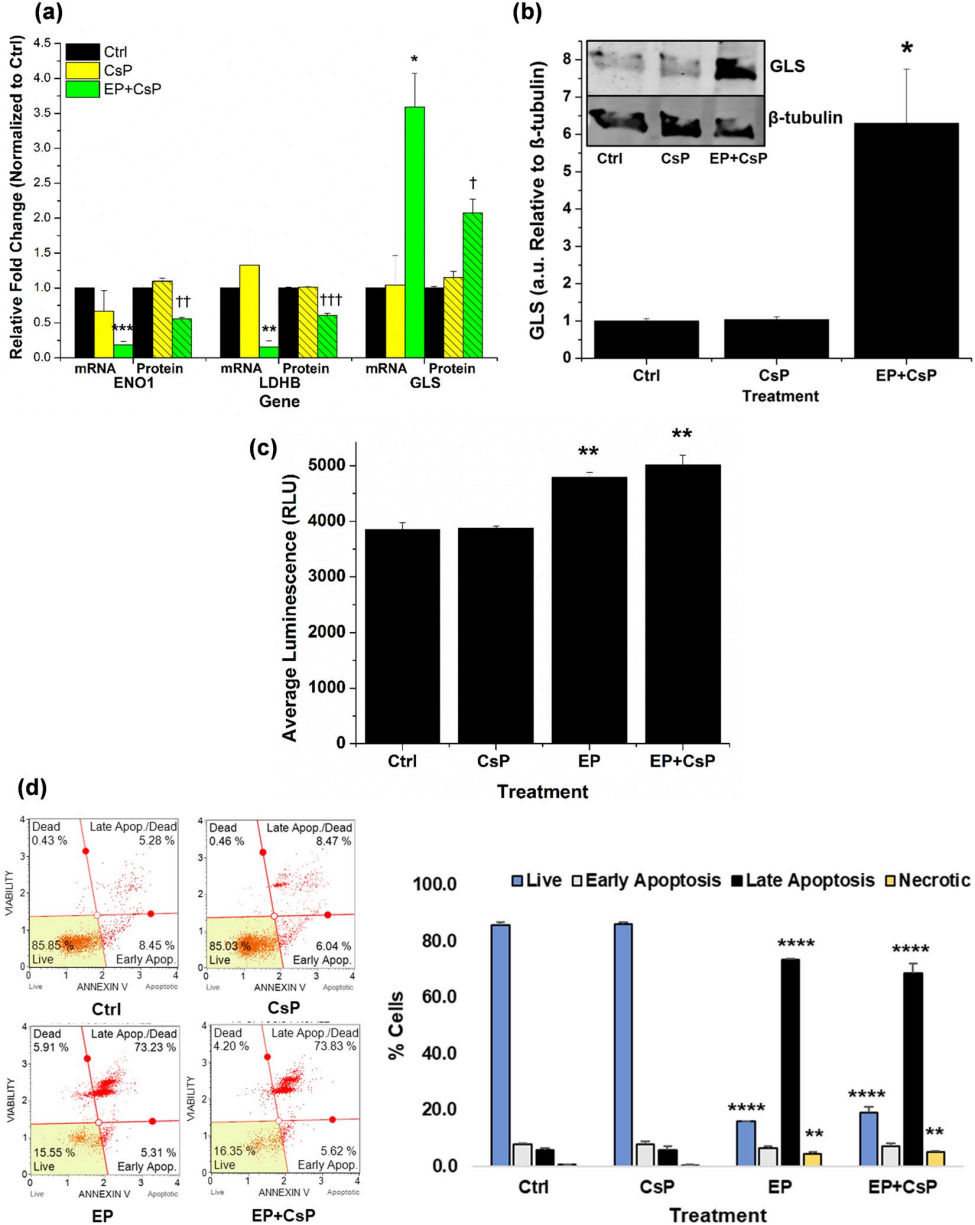
The mRNA and protein level expressions of enolase 1 (ENO1), lactate dehydrogenase (LDHB) and glutaminase (GLS) in CsP and EP + CsP treatments compared to the Ctrl. The ∆∆Cq method was used to determine relative mRNA expression levels at 4 h of the treatment from qPCR data with glyceraldehyde 3-phosphate dehydrogenase (GAPDH) as an internal reference. The protein expression levels were recorded at 4 h of the treatment using proteomics (N = 3). *P < 0.05, **P < 0.005-mRNA levels significantly different from Ctrl. ^†^P < 0.05, ^††^P < 0.005, ^†††^P < 0.0005-protein levels significantly different from Ctrl. (**b**) Validation of GLS protein level expression. Bars show the quantified expression of GLS in MDA-MB-231 cells under different treatments at 4 h. Representative western blot (WB) is shown in the inset, the WB signals were normalized with β-tubulin and were reported relative to the Ctrl (N = 3). *P < 0.05-protein levels significantly different from Ctrl. The full-length blots are presented in Supplementary Fig. S2. (**c**) The generation of H_2_O_2_ at 4 h (N = 5). **P < 0.005-H_2_O_2_ levels significantly different from Ctrl. (**d)** The apoptosis profile at 4 h. The apoptosis was assessed by incubating cells for 20 mins at RT with Annexin-V/7-Aminoactinomycin D (7-AAD) Reagent, which was followed by measurement of differential Annexin-V/7-AAD staining using a Muse^®^ Cell Analyzer. Significant difference between EP + CsP treated cells and Ctrl cells is indicated as **P < 0.005, ****P < 0.00005. Error bars are calculated using standard error.

To determine whether changes in the protein levels correspond to the changes at the transcript levels, we performed real time quantitative PCR (qPCR) experiments after 4 h of treatment for Ctrl, CsP and EP + CsP samples. Figure 7a shows mRNA level expressions of ENO1, LDHB, and GLS genes at 4 h of the treatments, and the comparison of the protein level changes observed at 4 h. The mRNA/protein levels were normalized with mRNA/protein expression levels of the Ctrl (level 1). The mRNA levels of ENO1 and LDHB genes decreased to 0.19 and 0.15 in EP + CsP as compared to the Ctrl, while they were 0.67 and 1.33 for CsP. In comparison, the protein expression of ENO1 and LDHB genes in EP + CsP decreased to 0.56 and 0.61 from the Ctrl, while they were 1.10 and 1.01 for CsP. Though we observed some minor up/down regulation in the mRNA levels of ENO1 and LDHB genes for CsP compared to Ctrl, no statistically significant difference was found, as also observed at the protein level. This validates our proteomics results and suggests that downregulation of ENO1 and LDHB is regulated at the transcript levels for EP + CsP. On the other hand, we observed ~3.5-fold increase in the mRNA level expression of GLS in EP + CsP and CsP, while it also increased by 2.08-fold from Ctrl and 1.8-fold from CsP, at the protein level. Overall, the mRNA level expressions of ENO1, LDHB and GLS correlate well with the protein level expressions.

We further validated the expression levels of GLS using the immunoblotting. Figure 7b shows the quantification of GLS expression for different treatments, normalized with β-tubulin and reported relative to the control. The representative immunoblots are shown in the inset. We observed that the GLS expression levels were ~1 for Ctrl and CsP treatments, not significantly different from each other. The EP + CsP treatment caused a 6-fold increase in the GLS expression levels from the control and CsP. This increase in GLS level in the immunoblotting correlates with the mRNA and protein level expressions for EP + CsP.

In addition, the reactive oxygen species (ROS) production levels in MDA-MB-231 cells were investigated following different treatments. Figure 7c shows the average luminescence (Lum) as relative light units (RLU), which directly corresponds to H_2_O_2_ levels in MDA-MB-231 cells at 4 h. We observed a marginal increase in Lum to 3873 for CsP from 3852 in the Ctrl, suggesting that CsP alone did not cause a significant oxidative stress in TNBC cells at 4 h. The Lum was 4789 for EP and it was 5021 for EP + CsP, a 1.2 to 1.3-fold increase in H_2_O_2_ levels compared to the cells treated with Ctrl and CsP. These results indicate that the EP + CsP and EP treatments increase ROS production in TNBC cells to cause oxidative stress inducing the cell death.

Cell death may take the form of apoptosis, a controlled and programmed cell death, or necrosis, a toxic and inflammatory process where the cell is a passive victim causing the expulsion of cellular constituents into the extracellular environment^37^. To understand the manner of cell death due to EP + CsP treatment, we stained the cells with Annexin V/7-Aminoactinomycin D (Annexin-V/7-AAD) and performed flowcytometry for quantitative analysis of live, early and late apoptosis, and necrotic cells for different treatment conditions (Fig. 7d). The EP and EP + CsP treatments reduced the fraction of live cells to 16 and 19% from 86% in Ctrl or CsP. The fraction of cells in the late apoptosis phase increased significantly to 69% from 6% in Ctrl and CsP. There was also an increase of necrotic cell population to 5% from 0.5% in the Ctrl and CsP. We observed that in EP + CsP, a total of 76% of cells were going through apoptosis, compared to only 5% undergoing necrosis. Similar behavior was also observed for the EP treated cells, where the apoptosis was prominent than necrosis with 73% of cells in late apoptosis, compared to 4% in necrosis. This indicates that EP + CsP and EP treatments induce apoptosis in majority of MDA-MB-231 cells, while causing necrosis in minority cell population to cause cell death. Thus, EP with and without CsP increases the production of ROS to cause oxidative stress and induces the apoptosis.

## Discussion

The EP + CsP treatment altered the proteome landscape of MDA-MB-231 cells, compared to control, CsP, and EP only treatments. These cells are relatively resistant to commonly used chemotherapeutics (Cisplatin, Paclitaxel, and Doxorubicin)^38^, and are a standard *in vitro* model of highly invasive TNBC. The EP + CsP treatment caused effective cell death, while affecting cellular metabolism. The EP alone also compromised cell viability, and metabolic activity in these cells. However the cell viability recovered for EP only treatment. With EP + CsP treatment, the effects were long-lasting, and the cells remained metabolically inactive, indicating the superiority of the EP + CsP treatment. We also showed that the effects of EP + CsP are specific to TNBC cells as they do not affect non-tumorigenic mammary epithelial MCF10A cells, as much.

The proteomics results showed that EP + CsP affected not only the membrane proteins, but also proteins in multiple components, biological functions and processes demonstrating a cell-wide effect. The most prominent shift in cellular metabolism was observed for glycolysis, TCA cycle and OXPHOS. Glucose metabolism has been shown to influence proliferation and differentiation of cancer cells^39^. We observed downregulation of key glycolysis pathway proteins but upregulation of TCA cycle. Similar effects were also observed for EP. Eleven and fourteen of the 28 glycolytic enzymes identified in this study were downregulated in EP + CsP compared to Ctrl and CsP treatments. However, only twelve glycolytic enzymes were downregulated for EP compared to CsP and there was no enrichment of the glycolytic pathway compared to control.

The various key proteins of glucose metabolism that were downregulated include ACSS2, ALDOA, ENO1, PGK1, and LDH isoforms-LDHA and LDHB, for EP + CsP and EP. The expression of ACSS2 was reported to be inversely correlated with survival in a cohort of 154 cases of TNBC, indicating that it may be an effective anticancer target^40^. The inhibition of ALDOA was shown to break the feed-forward loop of glycolysis to inhibit the proliferation of cancer cells^41^. The ENO1 is a potential biomarker of TNBC, and its function in glycolysis is consistent with the distinct TNBC metabolism compared to other breast cancer subtypes^42,43^. The PGK1 is elevated in breast cancer tissues compared to normal tissues, and it’s expression is dependent on the oxygen tension^44^. It has been shown to affect DNA replication and is found to be overexpressed in CsP-resistant ovarian cancer^45^. The LDH enzyme subunits A and B can catalyze the forward and backward conversion of pyruvate to lactate. The LDHA is an indicator of the breast cancer malignancy degree and its inhibition is shown to prevent TNBC brain metastasis^46^. The LDHB is shown to be specifically upregulated in basal-like/TNBC cell lines and tumors compared to luminal subtypes, and its expression results in a more comprehensive shift in TNBC tumor metabolism^47^. The increased expression of LDHB correlates to significantly poor clinical outcomes and is an essential gene for TNBC proliferation and survival *in vitro* and *in vivo*^47,48^. Recent reports suggest a novel, alternate hypothesis on metabolism in tumor cells, where hypoxic and glucose-deprived cells depend upon LDHB to utilize lactate secreted from the adjacent cells, which are undergoing aerobic glycolysis, in a phenomenon called “reverse Warburg effect”^47,49^. LDHB in tumor cells utilize lactate as energy source by converting it to pyruvate^50,51^, which feeds into the TCA cycle, indicating that the ECT of cells may also inhibit the reverse Warburg effect by downregulating LDHB expression. Considering this, the TNBC cells, which are distinctly wired for the dependence on glucose metabolism, are highly susceptible for its intervention^47^.

The ENO1 and LDHB mRNAs were downregulated in EP + CsP suggesting that the key glycolytic enzymes are regulated at the transcription level, which translates to proportional changes observed at the protein level. Increased expression in GLS at the mRNA level and protein level also appears to be consistent with the metabolic shift observed in this study. GLS converts glutamine to glutamate to generate α-ketoglutarate (αKG), which serves as a fuel to the TCA cycle.

While glycolysis was down, we observed upregulation of TCA cycle and OXPHOS pathway, indicating a switch in the metabolism from glycolysis to cellular respiration, with a larger dependency on oxidative energy substrates for energy production for TNBC cells treated with EP + CsP and EP. The increased OXPHOS could also be attributed to the stress response as the cells try to generate ATP through alternate sources when the glycolysis is down. We observed an upregulation in several pathways, such as fatty acid degradation, fatty acid metabolism, fatty acid elongation, tryptophan metabolism, amino acid metabolism, glyoxylate and dicarboxylate metabolism which can produce pyruvate and TCA cycle intermediates like acetyl COA and oxaloacetate to replenish the TCA cycle. The increase in mitochondrial metabolic activities (OXPHOS and TCA Cycle) in response to the EP may enhance CsP induced cytotoxicity, as observed for the EP + CsP^52^.

Moreover, we observed an upregulation of peroxisome proliferator-activated receptor (PPAR) pathway proteins. The expression of long chain fatty acid transport protein 4 (SLC27A4/FATP4), which transports long-chain fatty acid through the membrane was upregulated for EP + CsP, compared to CsP. This indicates that EP application may increase cellular transport, not only through the membrane pore formation, but also via the upregulation of key transport proteins. The role of PPAR pathway is not only limited to the transport of the fatty acid, but it can also promote fatty acid degradation and fatty acid metabolism, which in turn can promote acetyl COA synthesis to replenish the TCA cycle.

The upregulation in the peroxisome pathway may also be linked to the TCA cycle, as it involves breaking down of the fatty acid to fuel the TCA cycle and to generate the cell membrane. Since, electroporation is a membrane phenomenon, the increased activity of the peroxisome pathway can also be towards the efforts to repair or regenerate the membrane following ECT. In addition, peroxisome pathway is involved to generate H_2_O_2_ and to activate apoptosis^53^, correlating our results with previous studies^54,55^.

We also observed an upregulation of the calcium signaling pathway proteins only in EP + CsP treated cells from CsP, including the expression of voltage dependent anion channel proteins 1, 2, and 3 (VDCA1, VDCA2, VDCA3) and stromal interaction molecule 1 (STIM1). The VDCA channel proteins facilitate the diffusion of metabolites, ions (including calcium ions) and small hydrophilic molecules across outer mitochondrial membrane and regulate metabolism and mitochondria functions^56^. Also, VDAC1 expression increases with the increase in intracellular calcium concentration in response to apoptosis inducing agents^57^. STIM1 also works as a calcium sensor and is identified to be involved in calcium influx into the cells from the extracellular environment^58^. These proteins were specially affected for the EP + CsP, not for EP, indicating that ECT with cisplatin can facilitate the calcium transport into the cells, which is consistent with earlier finding, where they also used 100 µs pulses^59^. Calcium release from endoplasmic reticulum (ER) also causes the redistribution of the ER calcium sensor STIM1^60^. This links very well with the observed significant upregulation in protein processing in the ER pathway, which happens in response to the ER stress. It is previously shown that under stimulation, several unfolded and incompletely folded proteins accumulate in the ER lumen to trigger unfolded protein response (UPR), leading to ER stress and apoptosis^61^. This indicates that in the cells treated with EP + CsP, the increased intracellular calcium concentration due to the calcium influx from extracellular environment and its release from ER, can trigger apoptosis, under conditions with high ROS production^62^. This also correlate very well with the previous observations, where microsecond electrical pulses released calcium from the ER and other internal organelles^63^.

To confirm these observations, we further evaluated the H_2_O_2_ levels and the apoptosis profile. We showed that the ROS production increases significantly in response to the EP + CsP and EP treatments, which may cause the oxidative stress in these cells to induce apoptosis. This correlates very well with our proteomics results as well as those reported previously by other researchers, who have shown that EP application can generate ROS, which can activate the apoptosis signaling pathways in cells^54,55,64,65^.

However, cancer cells harbor inherent resistance to ROS induced apoptosis^66^, which can significantly compromise the effectiveness of this EP-based therapy. Compared to EP, EP + CsP downregulated SLC1A5, which may sensitize MDA-MB-231 to ROS induced apoptosis^66^, and lead to a sustained effect of EP + CsP, as observed in this study. The RFC4, which promotes DNA double stranded break repair to cause resistance^67^, was also downregulated for EP + CsP from EP, indicating that DNA repair is slower in EP + CsP treated cells. In addition, the expression of G3BP2, which is implicated in the maintenance of breast tumor-initiating cells (TICs)^68^, was downregulated for EP + CsP, compared to EP. These results highlight the differences in the protein expression profiles for EP + CsP and EP treatments, and suggest that the effects of EP + CsP treatment were more comprehensive, compared to EP.

In summary, these novel findings indicate that EP + CsP can alter the metabolic profile of the MDA-MB-231 cells to effectively target them by utilizing their glycolytic vulnerabilities and inducing apoptosis utilizing the synergy of multiple pathways.

## Methods

### Cell culture

The MDA-MB-231, human adenocarcinoma epithelial TNBC cells (ATCC^®^, USA) and MCF10A, human non-tumorigenic mammary epithelial cells (ATCC^®^) were studied.

The MDA-MB-231 were grown and maintained at 37 °C, 70–80% humidity, and 5% CO_2_ in Dulbecco’s Modified Eagle Medium (DMEM) (Gibco™, USA) supplemented with 1% Penicillin-Streptomycin and 10% FBS (Corning™, USA).

The MCF10A were grown and maintained at 37 °C, 70–80% humidity, and 5% CO_2_ in DMEM:F12 (1:1) supplemented with 5% horse serum (Atlanta Biologicals), 20 ng/ml human EGF (Sigma-Aldrich, USA), 0.5 mg/mL hydrocortisone (Sigma-Aldrich), 100 ng/mL cholera toxin (Sigma-Aldrich), 10 mg/mL bovine insulin (Sigma-Aldrich), 100 IU/mL penicillin and 100 mg/mL streptomycin.

For treatment, cells were detached using trypsin, were centrifuged at 1000 rpm for 5 min at 4 °C and were resuspended in fresh media at 1 × 10^6^ cells/mL.

### Drug

Cisplatin’s (Sigma-Aldrich) stock solution was prepared by dissolving it in sterile double-distilled water. The required volume from Cisplatin stock was added into cells to make 100 μM Cisplatin treatment concentration.

### Electrical pulse application

Electroporation was performed using the BTX-ECM830 electroporator (Genetronics Inc., USA). Eight, unipolar, square-wave pulses of 1 Hz frequency at 1200 V/cm with 100 μs pulse duration were applied to 600 μL cells, suspended at 1 × 10^6^ cells/mL in fresh-media with Cisplatin in BTX electroporation cuvettes (4 mm gap). Control and Cisplatin treatment did not receive any electrical pulse. Following treatment, cells were transferred to 6-well plates (600,000 cells/well) containing 2 mL of fresh-media and were cultured for 4 h for proteomics, flow cytometry, RT-qPCR, and immunoblotting experiments.

### Flowcytometric assessment of cell viability and apoptosis

Following 4 h treatment, all cells were detached using trypsin, centrifuged, and were resuspended in fresh media, after the removal of supernatant. For cell viability, suspended cells (20 μL) were stained with 380 μL Muse™ Count & Viability Reagent (EMD Millipore, USA). For apoptosis, suspended cells (100 μL) were stained with 100 μL Muse^®^ Annexin V Dead Cell Assay kit (EMD Millipore). Following staining, samples were incubated in dark and were run on Muse^®^ Cell Analyzer (EMD Millipore) as per the instrument’s protocol.

### Cell metabolic activity assessment

After treatment, cells were transferred to 96-well plates (20,000 cells/well) containing fresh-media and were cultured for 24 h to assess the metabolic activity using RealTime-GloTM^®^ MT Cell Viability kit (Promega, USA), as per manufacturer’s protocol. At 4 h and 24 h, luminescence (Lum) was measured at 1 sec integration using a SpectraMax M5 spectrophotometer (Molecular Devices, USA). The Lum of control was used to normalize sample Lum to determine metabolic activity at 4 h or 24 h by Eq. (1).

1 $$Cell\,Metabolic\,Activity({\rm{ \% }})=\frac{{\rm{L}}{\rm{u}}{\rm{m}}\,{\rm{v}}{\rm{a}}{\rm{l}}{\rm{u}}{\rm{e}}\,{\rm{o}}{\rm{f}}\,{\rm{s}}{\rm{a}}{\rm{m}}{\rm{p}}{\rm{l}}{\rm{e}}}{{\rm{L}}{\rm{u}}{\rm{m}}\,{\rm{v}}{\rm{a}}{\rm{l}}{\rm{u}}{\rm{e}}\,{\rm{o}}{\rm{f}}\,{\rm{c}}{\rm{o}}{\rm{n}}{\rm{t}}{\rm{r}}{\rm{o}}{\rm{l}}\,{\rm{a}}{\rm{t}}\,4\,{\rm{h}}}\times 100$$

### Assessment of hydrogen peroxide (H_2_O_2_) ROS

After treatment, cells were transferred to 96-well plates (20,000 cells/well) with 60 μL fresh-media and 20 µL of H_2_O_2_ substrate solution from ROS-Glo^TM^ H_2_O_2_ assay kit (Promega) and were cultured for 4 h. ROS-Glo™ detection solution was added to cells and incubated for 20 min, and Lum was recorded for 0.5 sec integration using the SpectraMaxM5 spectrophotometer.

### Real time quantitative PCR

Following 4 h treatment, all cells were trypsinized and washed thrice with ice-cold 1 × PBS. RNeasy Mini Kit (QIAGEN, USA) was used as per manufacturer’s protocol to extract the RNA. RNA was quantified and cDNA was synthesized. The cDNA was then used in CFX Connect™ RT-PCR system (Bio-Rad Laboratories, USA) to measure mRNA levels. The relative mRNA levels were determined using the ∆∆Cq technique using Glyceraldehyde 3-phosphate dehydrogenase (GAPDH) as the internal reference. The following conditions were used: 10 min at 95 °C, [15 sec at 95 °C, 10 sec at 60 °C, 30 sec at 72 °C] × 45 cycles, 10 sec at 95 °C, 60 sec at 65 °C, 1 sec at 97 °C. The primer sequences were; Enolase1 (ENO1): upstream 5′-AAAGCTGGTGCCGTTGAGAA-3′, downstream 5′-GGTTGTGGTAAACCTCTGCTC-3′. Lactate Dehydrogenase (LDHB): upstream 5′-CCTCAGATCGTCAAGTACAGTCC-3′, and downstream 5′-ATCACGCGGTGTTTGGGTAAT-3′. Glutaminase (GLS): upstream 5′-TCTACAGGATTGCGAACGTCT-3′, and downstream 5′-CTTTGTCTAGCATGACACCATCT-3′. GAPDH: upstream 5′-ACAACTTTGGTATCGTGGAAGG-3′ and downstream 5′-GCCATCACGCCACAGTTTC-3′.

### Immunoblotting

Following 4 h treatment, all cells were scraped, washed thrice with ice-cold 1 × PBS with centrifugation at 1,500 rpm at 4 °C, lysed using the RIPA buffer and sonication, as previously^69^. Lysates were cleared of debris with centrifugation at 14000 rpm. The Pierce^®^ BCA Assay (Thermo Fisher Scientific, USA) was used to estimate the protein concentration. The 5 × sample-buffer was added into protein (20 μg) from sample and heated to denature the protein. SDS-polyacrylamide gel electrophoresis was performed to separate the denatured proteins and were transferred onto a polyvinyl difluoride (PVDF) membrane for antibody staining and protein detection. The PVDF membrane was incubated overnight with 5% (w/v) nonfat dry milk at 4 °C for blocking. The blocked membrane was probed for proteins using primary antibodies for GLS (rabbit, PA5-35365; Thermo Fisher Scientific) and β-tubulin (mouse, E7; DSHB, University of Iowa). The proteins were detected using the appropriate Alexa Fluor^®^ secondary antibody (anti-rabbit, A-21109 or anti-mouse, A11375; Thermo Fisher Scientific). The blots were imaged at 680 nm for GLS and 790 nm for β-tubulin using the Li-Cor Odyssey infrared imaging System and were quantified using ImageJ.

### Sample preparation for mass spectrometry analysis

Following 4 h treatment, all cells were scraped, washed thrice in ice-cold 1 × PBS with centrifugation at 1,000 rpm at 4 °C, and were re-suspended in 4 M urea. Sample preparation was done, as previously^70^. In brief, cells were homogenized in Precellys^®^ 24 Bead Mill Homogenizer (Bertin Corp., USA) and were centrifuged at 14,000 rpm for 15 min at 4 °C. Supernatant proteins were incubated overnight at −20 °C with pre-chilled (−20 °C) acetone (five equivalents (v/v)) for precipitation. Precipitated protein pellets were dissolved in 8 M urea and protein concentration was estimated using BCA assay. Protein (50 µg) from each sample was reduced with 10 mM dithiothreitol (DTT) at 55 °C for 45 min followed by cysteine alkylation with 20 mM iodoacetamide at room temperature under dark for 45 min and an additional 5 mM DTT for 20 min at 37 °C. Proteins were digested overnight at 37 °C with Trypsin/Lys-C Mix (Promega) enzyme-protein at ratio 1:25 (w/w) and were passed through C18 micro spin columns (The Nest Group Inc., USA). Peptides elution was performed using 0.1% formic acid (FA) in 80% acetonitrile (ACN). Eluted peptides were vacuum dried and were re-suspended in 0.1% FA in 3% ACN. BCA assay was used to estimate peptide concentration and the concentration was adjusted to 0.5 µg/µL.

### LC-MS/MS data collection

LC-MS/MS data were collected using a reverse-phase HPLC-ESI-MS/MS system by coupling an UltiMate^TM^ 3000 RSLCnano to a Q-Exactive (QE) High Field (HF) Hybrid Quadrupole Orbitrap^TM^ MS (Thermo Fisher Scientific) and a Nano-spray Flex^TM^ ion source (Thermo Fisher Scientific). Samples were analyzed using a standard data-dependent acquisition mode. Mobile phase solvent A was a 98% purified water/2% ACN/0.01% FA, and solvent B was 80% ACN/20% purified water/0.1% FA. Peptides (1 µg) were loaded onto a trap column (300 µm ID × 5 mm, 5 µm 100 Å PepMap C18 medium) at 5 µL/min flow rate. After 5 min, trap column was switched in-line with Acclaim™ PepMap™ RSLC C18 (75 µm × 15 cm, 3 μm 100 Å PepMap C18 medium, Thermo Fisher Scientific) analytical column and peptide were separated using 120 min LC gradient method at 35 °C. A 5–30% linear gradient of solvent B was run for 80 min, followed by 11 min of 45% solvent B and 2 min of 100% solvent B with an additional 7 min of isocratic flow. Solvent A was then applied at 95% for 20 min for column equilibration. A Top20 data-dependent MS/MS scan method was used to acquire MS data. Injection time was set to 100 milliseconds, resolution to 120,000 at 200 m/z, spray voltage of 2 eV and an AGC target of 1 × 10^6^ for a full MS spectra scan with a range of 400–1650 m/z. High-energy C-trap dissociation was used to fragment precursor ions at a normalized collision energy of 27 eV. The MS/MS scans were acquired at a resolution of 15,000 at 200 m/z. The dynamic exclusion was set at 30 sec to circumvent repeated scanning of identical peptides.

### Data analysis

MaxQuant (v1.6.1.0)^71,72^ was used to process MS/MS data against the Uniprot *Homo sapiens* fasta (http://www.uniprot.org; 26,446 entries as of October 05, 2018) concatenated with a common contaminants and a reverse-decoy database, as previously^73^. Cleavage enzymes were setup as Trypsin/P and LysC, with toleration of up to 2 missed cleavages. Mass error was set to 10 ppm and 20 ppm for precursor and fragment ions, respectively. Cysteine alkylation and methionine oxidation was set to fixed and variable modifications, respectively. The 0.01 was estimated false discovery rate threshold for both the peptides and the proteins levels. The peptide quantitation was conducted using “unique plus razor peptides”. The non-unique peptides assigned to protein/protein group with most other peptides were razor peptides. Following MaxQuant processing, proteins with no MS/MS count and zero LFQ intensity were removed. The results were transferred to Data Analysis and Extension Tool (DAnTE) for Pearson correlations analysis. Proteins with LFQ≠0 and MS/MS ≥2 in at least two replicates for ≥1 of the treatments were retained for further analyses using a MATLAB script. Zero LFQ values were imputed with 281660, half of the lowest LFQ value (563320) observed across three treatments. LFQ intensities were log2 transformed and average was calculated for three replicates. The fold-change was calculated by subtracting the average log2 values [Δlog2 (LFQ intensity)] between proteins from each comparison group (CsP vs Ctrl; EP vs Ctrl; EP+CsP vs Ctrl; EP+CsP vs EP; EP vs CsP; EP+CsP vs CsP). Proteins with fold-change of |Δlog2| > 0.5, and P < 0.05 (Student’s unpaired, two-tailed, t test) were significantly regulated.

### Enrichment and string interaction analysis

Significantly regulated proteins were compared against the background of total 2867 quantified proteins to discover the pathway enrichment using KEGG database in DAVID 6.8^74^. Cellular localization and molecular functions of differentially expressed proteins was performed using PANTHER Classification System^75^ against *Homo sapiens* database. STRING^76^ was used to visualize the interaction and functional enrichment with minimum required interaction score as medium confidence (0.4) and kmeans clustering (3 clusters).

### Statistical analysis

Repeated Measure ANOVA was used to derive statistical significance for cell metabolic activity, coupled with Tukey’s multiple comparison test. MDA-MB-231 metabolic activity data was log transformed prior to statistical analysis to satisfy normality and homoscedasticity assumptions. Tukey’s test tags each treatment with a letter or a group of letters to indicate their significance. The same letter or the same groups of letters indicate that they are not significantly different. The different letters or different group of letters indicate that they are significantly different (P < 0.05).

Student’s unpaired, two-tailed, t-test was used for testing significance for log2 transformed proteomics data, and all other experimental data.

All experiments were performed in triplicates.

## Supplementary information

Supplementary Information

Table S1, Table S2, Table S3, Table S4, Table S5, Table S6

## Data Availability

RAW proteomics data files, parameters used, and LC–MS/MS methodology and statistics have been deposited in the MassIVE public proteomics data repository (MassIVE ID: MSV000083360). The datasets generated and/or analyzed during the current study are available from the corresponding author on reasonable request.
