# Author Correction: Quantitative proteomic analysis of enhanced cellular effects of electrochemotherapy with Cisplatin in triple-negative breast cancer cells

**DOI:** 10.1038/s41598-019-55880-7

**Published:** 2019-12-10

**Authors:** Lakshya Mittal, Uma K. Aryal, Ignacio G. Camarillo, Rodrigo M. Ferreira, Raji Sundararajan

**Affiliations:** 1 School of Engineering Technology, Purdue University, West Lafayette, IN 47907 USA; 2 Purdue Proteomics Facility, Bindley Bioscience Center, Purdue University, West Lafayette, IN 47907 USA; 3 Department of Biological Sciences, Purdue University, West Lafayette, IN 47907 USA; 4 Purdue Center for Cancer Research, Purdue University, West Lafayette, IN 47907 USA

Correction to: *Scientific Report* 10.1038/s41598-019-50048-9, published online 26 September 2019

The Acknowledgements section in this Article is incomplete.

“L.M. is grateful to the partial support by the Purdue Polytechnic Institute Dean’s award GRA – FY16. Publication of this article was funded by Purdue University Libraries Open Access Publishing Fund. We thank Victoria Hedrick of the Purdue Proteomics Facility for support in LC-MS/MS data collection and for critical reading of the manuscript. All the mass spec experiments were performed at the Purdue Proteomics Facility, Bindley Bioscience Center. The Q Exactive Orbitrap HF and UltiMate 3000 HPLC system used for LC-MS/MS analysis were purchased from funding provided by the Purdue Office of the Executive Vice President for Research and Partnership. We thank Alexis A. Musleh for help in maintaining the cell cultures and assistance in experiments and Vishak Raman for useful discussions. The authors are grateful to Dr. Richard J. Kuhn’s lab for the use of spectrophotometer, Dr. Chun-Ju Chang’s lab for the Nanodrop^TM^, Dr. Ourania M. Andrisani’s lab and Dr. Emily C. Dykhuizen’s lab for their qPCR machines.”

should read:

“L.M. is grateful to the partial support by the Purdue Polytechnic Institute Dean’s award GRA – FY16. Publication of this article was funded by Purdue University Libraries Open Access Publishing Fund. We thank Victoria Hedrick of the Purdue Proteomics Facility for support in LC-MS/MS data collection and for critical reading of the manuscript. All the mass spec experiments were performed at the Purdue Proteomics Facility, Bindley Bioscience Center. The Q Exactive Orbitrap HF and UltiMate 3000 HPLC system used for LC-MS/MS analysis were purchased from funding provided by the Purdue Office of the Executive Vice President for Research and Partnership. We thank Alexis A. Musleh for help in maintaining the cell cultures and assistance in experiments and Vishak Raman for useful discussions. The authors are grateful to Dr. Richard J. Kuhn’s lab for the use of spectrophotometer, Dr. Chun-Ju Chang’s lab for the Nanodrop^TM^, Dr. Ourania M. Andrisani’s lab and Dr. Emily C. Dykhuizen’s lab for their qPCR machines. The authors are grateful to Prof. Morris Levy for his excellent edits.”

