# Supplementary material for: Quantitative proteomic analysis of enhanced cellular effects of electrochemotherapy with Cisplatin in triple-negative breast cancer cells
Source: Sci Rep. 2019 Sep 26;9:13916. doi: 10.1038/s41598-019-50048-9 (PMC6763474; doi:10.1038/s41598-019-50048-9)
Supplement: Supplementary file 1 — Supplementary Information [file 41598_2019_50048_MOESM1_ESM.docx]

**Quantitative proteomic analysis of enhanced cellular effects of electrochemotherapy with Cisplatin in triple-negative breast cancer cells**

Lakshya Mittal^1^, Uma K. Aryal^2,*^, Rodrigo M. Ferreira^3^, Ignacio G. Camarillo^3,4^, and Raji Sundararajan^1,*^

*^1^School of Engineering Technology, Purdue University, West Lafayette, IN 47907*

*^2^Purdue Proteomics Facility, Bindley Bioscience Center, Purdue University, West Lafayette, IN 47907*

*^3^Department of Biological Sciences, Purdue University, West Lafayette, IN 47907*

*^4^Purdue Center for Cancer Research, Purdue University, West Lafayette, IN 47907*

*****Corresponding authors **(**[raji@purdue.edu](mailto:raji@purdue.edu) and [uaryal@purdue.edu](mailto:uaryal@purdue.edu))

**Supplementary Information:**

***Supplementary Figures***


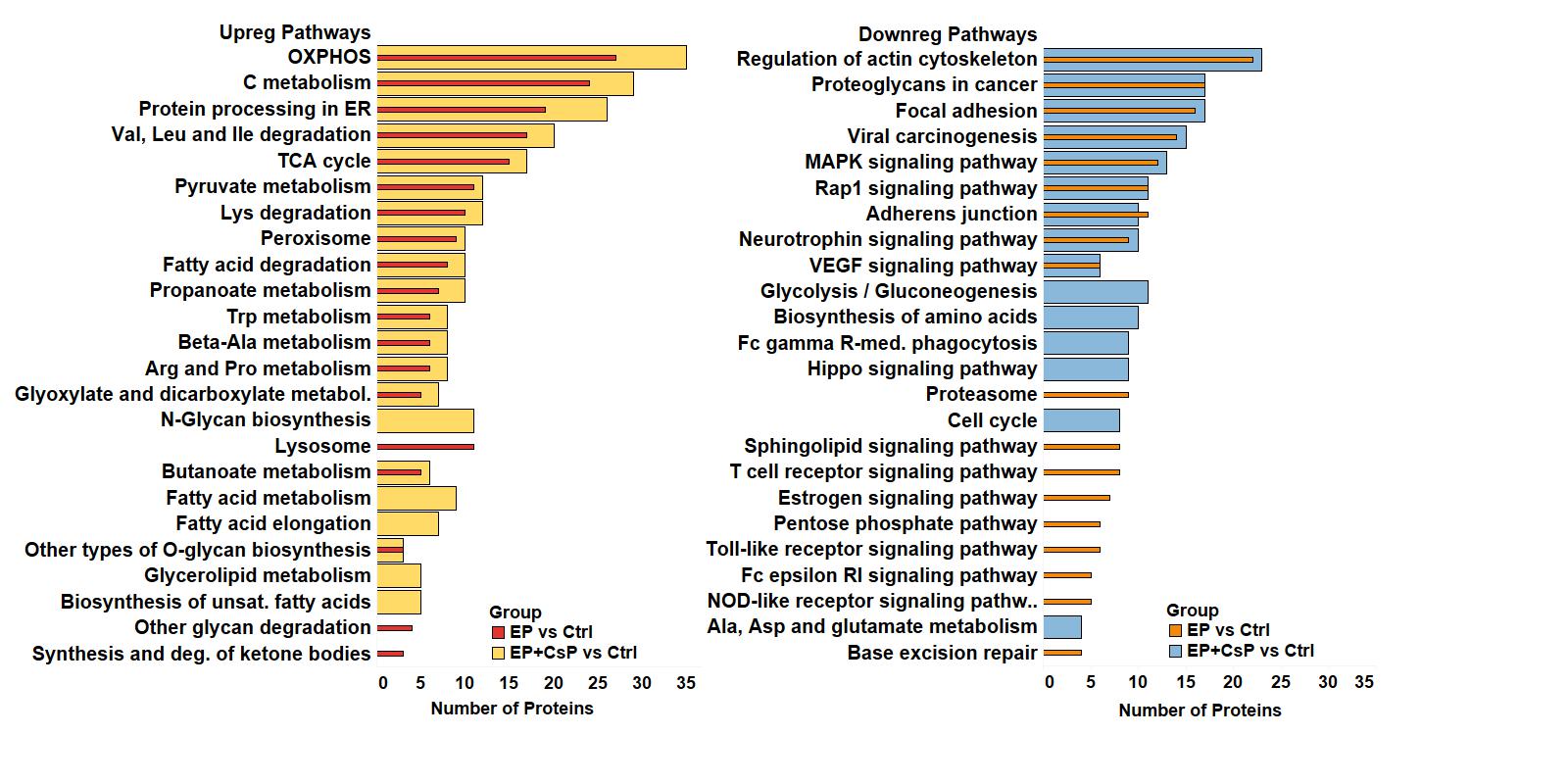

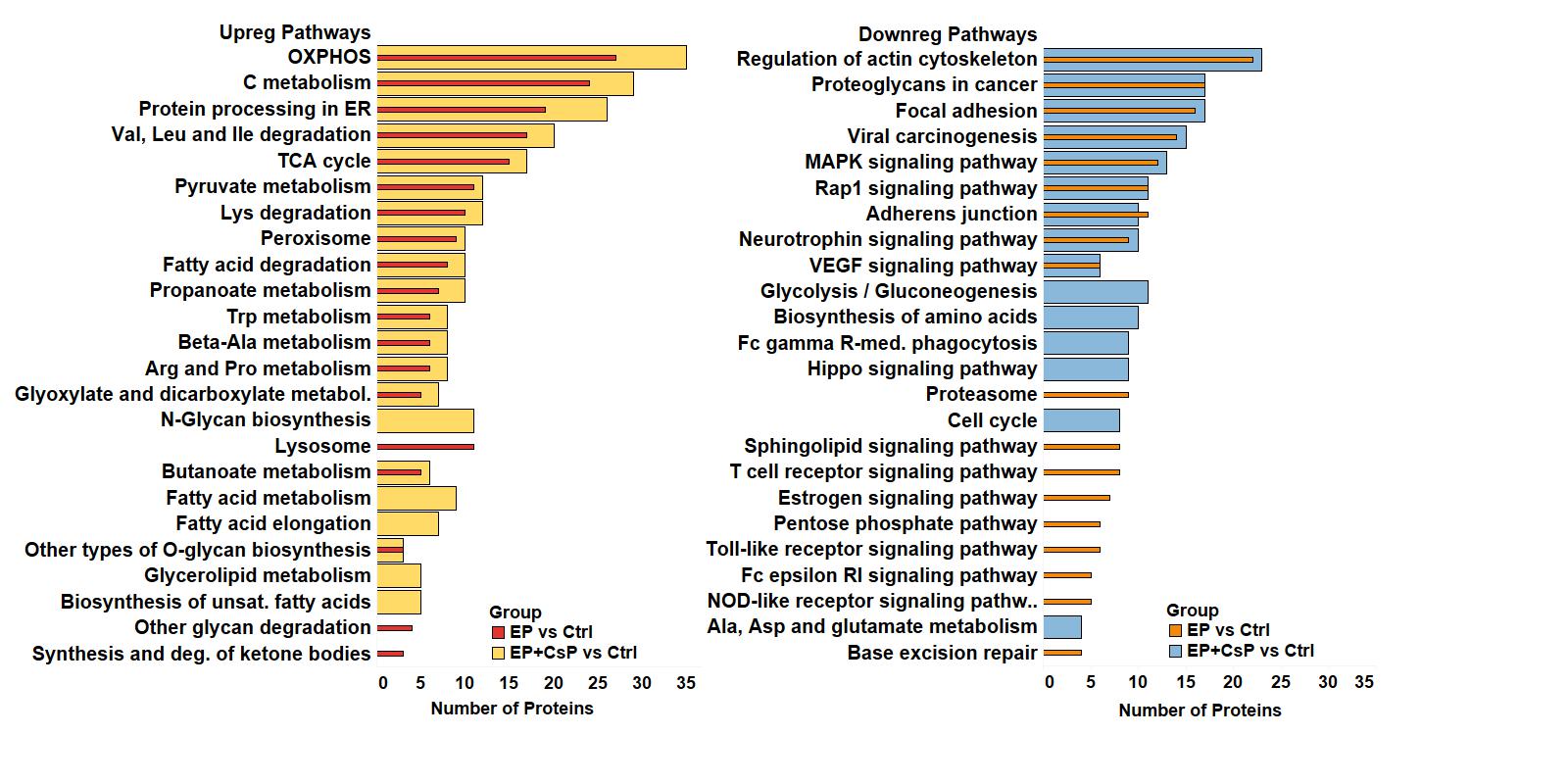

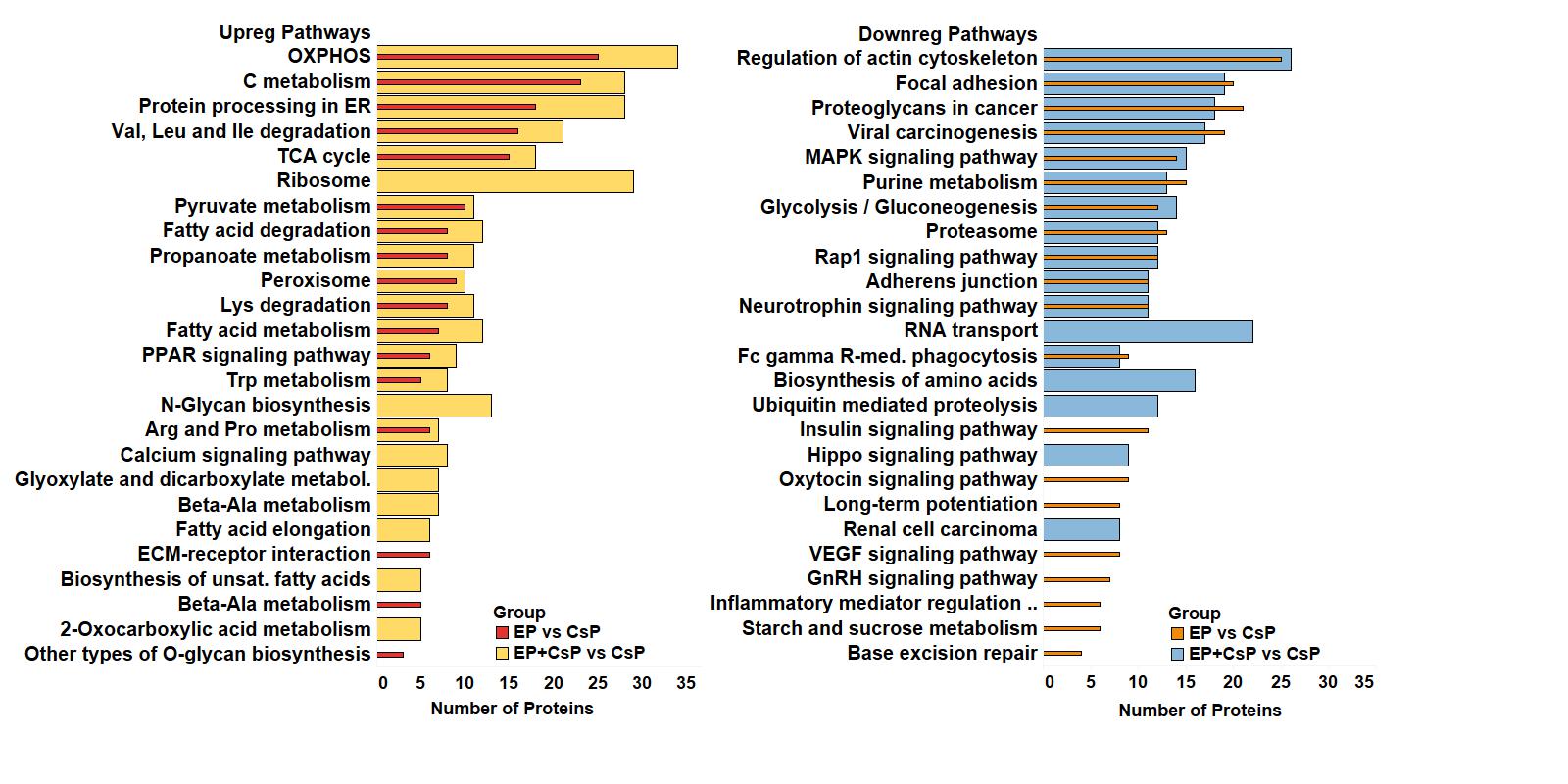

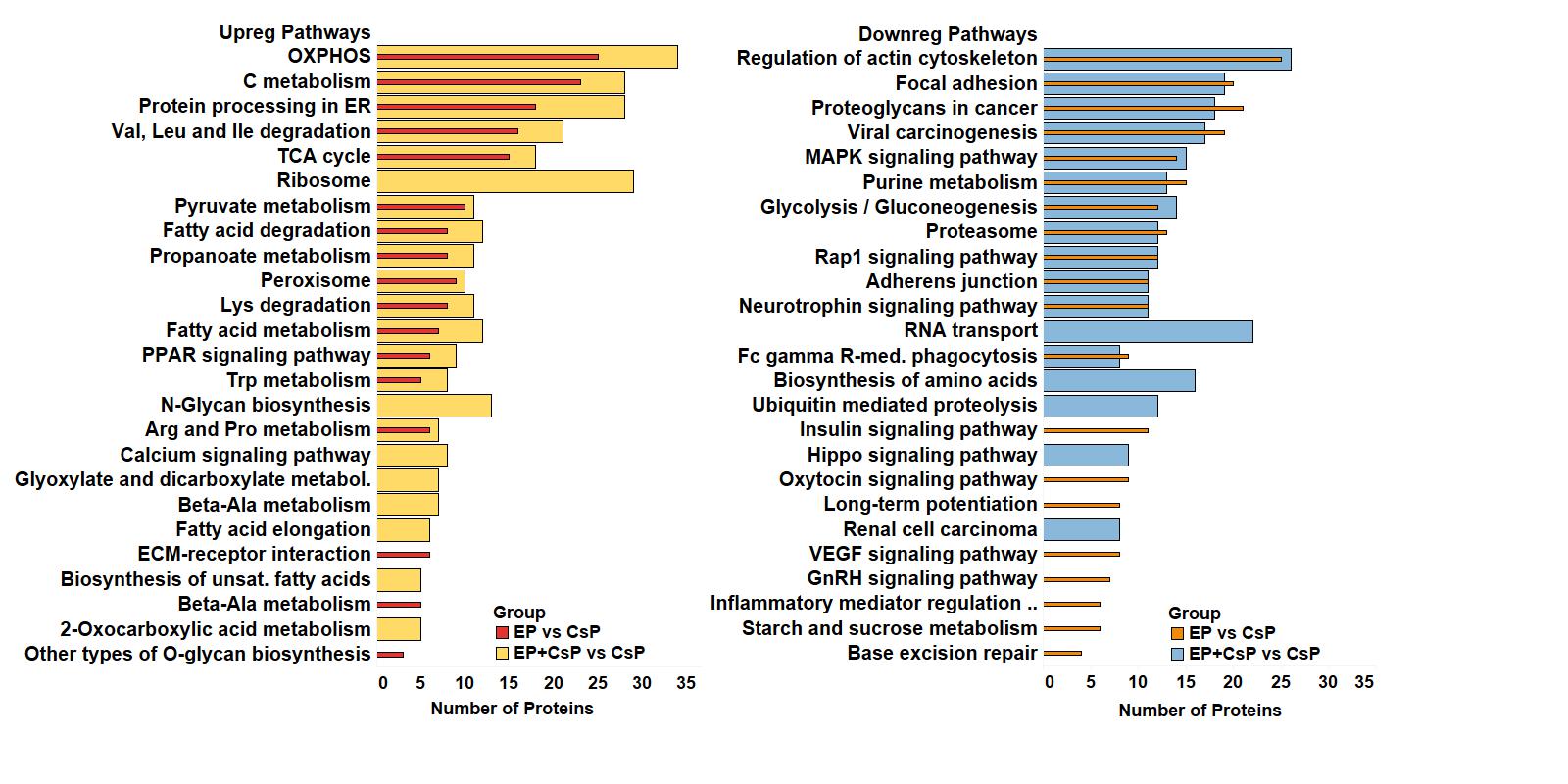


**(a)**

**(b)**

Figure S1: Enriched pathways for significantly upregulated (a) and downregulated (b) proteins using DAVID 6.8 in different pairwise comparisons: EP vs Ctrl, EP+CsP vs Ctrl, EP vs CsP, and EP+CsP vs CsP. Abbreviations-Ala: Alanine; Arg: Arginine; Asp: Aspartate; C: Carbon; Deg.: Degradation; Isoleucine: Ile; Leu: Leucin, Lys: Lysine; Pro: Proline; Trp: Tryptophan; Unsat: Unsaturated; Val: Valine.

Figure S2 shows the full-length blots probed for the Glutaminase (GLS) proteins (Figure S2a) with β-tubulin (Figure S2b) used as the loading control.


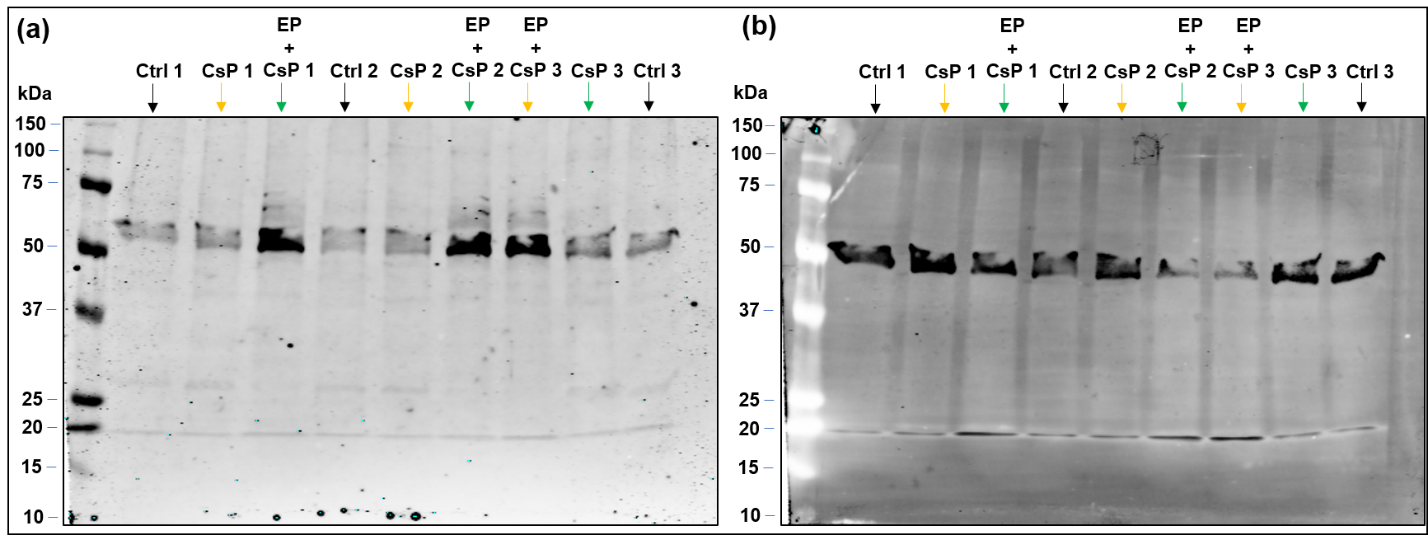


**Figure S2: Full-length western blot images used for Figure 6b in the manuscript: (a)** **Glutaminase (GLS). (b) β-tubulin, used as loading control. The same membrane was probed for proteins using primary antibodies for GLS (****rabbit, PA5-35365; Thermo Fisher Scientific) and β-tubulin (****mouse, DSHB, University of Iowa).** **The proteins were tagged using the secondary antibodies (anti-rabbit, A-21109 or anti-mouse, A11375; Thermo Fisher Scientific). The blots were imaged at 680nm for GLS and 790nm for β-tubulin and were converted to grey scale. The blots were imaged at 680nm for GLS and 790nm for β-tubulin and were quantified using ImageJ.**

***Supplementary Tables***

**Table S1: The metabolic activity (mean ± S.E.) of MDA-MB-231 cells up to 60h for different treatment conditions.**

**Table S2: Peptide sequence and raw intensity.**

**Table S3: Proteins identified in biological replicates are listed with their corresponding intensities and MS/MS (Spectral) counts.**

**Table S4: Table S4: Proteins which are identified to be significantly (p<0.05) regulated (|fold change|>0.5) in various pairwise comparisons: CsP vs Ctrl, EP+CsP vs CsP, EP+CsP vs Ctrl, EP+CsP vs EP, EP vs Ctrl, and EP vs CsP. The intensity is a log2 transformed LFQ intensity.**

**Table S5: Proteins which are identified to be significantly (p<0.05) regulated (|fold change|>0.5) for EP+CsP vs EP. The intensity is a log2 transformed LFQ intensity. Upregulated and downregulated proteins are represented with red and green colors, respectively.**

**Table S6: Fold change in glycolysis proteins which are identified to be significantly (p<0.05) regulated (|fold change|>0.5) in atleast one of the pairwise comparisons: CsP vs Ctrl, EP+CsP vs CsP, EP+CsP vs Ctrl, EP+CsP vs EP, EP vs Ctrl, and EP vs CsP. The fold change was calculated by normalizing the sample LFQ intensities with Ctrl LFQ intensity. Upregulated and downregulated proteins are represented with red and green colors, respectively.**
